# Environmental fungi and bacteria facilitate lecithin decomposition and the transformation of phosphorus to apatite

**DOI:** 10.1038/s41598-019-51804-7

**Published:** 2019-10-25

**Authors:** Chunkai Li, Qisheng Li, Zhipeng Wang, Guanning Ji, He Zhao, Fei Gao, Mu Su, Jiaguo Jiao, Zhen Li, Huixin Li

**Affiliations:** 1 College of Resources and Environmental Sciences, Nanjing Agricultural University, Nanjing, Jiangsu 210095 China; 2 Jiangsu Collaborative Innovation Center for Solid Organic Waste Resource Utilization, Nanjing, Jiangsu 210014 China; 3 Jiangsu Provincial Key Lab for Organic Solid Waste Utilization, Nanjing Agricultural University, Nanjing, 210095 China

**Keywords:** Element cycles, Environmental impact

## Abstract

Organophosphorus compounds (OP) are stable P source in nature, and can increase eutrophication risk in waterbodies. Lecithin was the most difficult OP to be broken down. In this study, two typical phosphate-solubilizing microorganisms, *Aspergillus niger* and *Acinetobacter* sp., were applied to evaluate their ability to decompose both inorganic phosphates and lecithin. *A. niger* and *Acinetobacter* sp. could solubilize calcium phosphates by secreting various organic acids, e.g., oxalic and formic acids. The fungus, *A. niger*, shows significantly higher ability of solubilizing these inorganic phosphates than *Acinetobacter* sp., primarily due to its secretion of abundant oxalic acid. However, the bacterium, *Acinetobacter* sp., could secrete more acid phosphatase than *A. niger* for lecithin decomposition, i.e., 9300 vs. 8500 μmol L^−1^ h^−1^. Moreover, after addition of CaCl_2_, the released P from lecithin was transformed to stable chlorapatite in the medium. To the contrast, Ca cations inclined to form calcium oxalate (rather than stable phosphate mineral) after the incubation of *A. niger*, as it induced relatively acidic environment after breaking down lecithin. Therefore, this work sheds light on the bright future of applying bacteria and Ca cations in OP pollutant management.

## Introduction

Phosphorus (P) derived from organophosphorus (OP) compounds constitutes 30 to 90% of the total P content in soil^1^. OP compounds are widely distributed in soil and appear as various forms, such as inositol phosphates, phospholipids, and nucleic acids^2^. However, most OP compounds in soil exist as insoluble forms, and only a small fraction (<1%) can be directly used by crops^3^. OP species, usually derived from the decomposition of aquatic organisms, increase the risk of eutrophication^4,5^. The efficient use of P and the remediation of P-induced eutrophication require a better understanding of biogeochemistry of OP compounds.

It is critical that phospholipids be transformed into soluble P, as OP compounds are more resistant to decay than are phytates and nucleic acids. The degradation of phospholipids to inorganic phosphate is regulated by phosphatases^6^, including two major types: phosphodiesterase (PDE) and phosphomonoesterase (PME)^7^. Many physical and chemical techniques have been applied to oxidise or break down phospholipids, including UV-LED/TiO_2_ photocatalytic/photoelectrocatalytic inactivation, acid-based hydrolysis, and graphene oxide-catalytic hydrolysis^8–10^. Soluble P originated from orthophosphates could also react with Pb^2+^, Ca^2+^, Mg^2+^, or Fe^3+^ cations to form minerals such as hydroxylapatite, struvite, or zwieselite, respectively^11–16^. However, the potential pathways of OP decomposition and P mineralization regulated by microorganisms are still poorly known.

Phosphate-solubilizing microorganisms (PSMs) are plant growth-promoting microorganisms that have traditionally been used to increase crop yields and improve nutrient levels in soil^17,18^. PSMs contribute to P release from both inorganic and organic matters. Solubilisation of (inorganic) phosphate involves the secretion of low molecular weight organic acids (LMWOAs) by PSMs^19,20^. The two major categories of PSMs are phosphate-solubilizing bacteria (PSB) and phosphate-solubilizing fungi (PSF)^21–23^. PSB include *Acinetobacter* sp., *Pseudomonas aeruginosa*, *Alcaligenes faecalis*, *Sphingobium* sp., *Pantoea agglomerans*, *Rahnella aquatilis*, *Enterobacter* sp., *Burkholderia cepacia*, and *Bacillus* sp.^24–28^. PSF also possess a remarkable ability to solubilise phosphate, including *Aspergillus niger*, *Penicillium*, *Curvularia*, and *Trichoderma*^29,30^. PSF usually have higher phosphate-solubilizing ability than PSB^31,32^. *A. niger* and *Acinetobacter* sp. function as the representative PSF and PSB, respectively^25,33,34^. Both *A. niger* and *Acinetobacter* synthesize and secrete phosphatase and phytase under P-deficient conditions, in addition to their secretion of organic acids^35,36^. However, few studies have examined the differences in the abilities to solubilize inorganic phosphates and break down OP compounds between PSB and PSF.

Lecithin, a model phospholipid, has amphiphilic and surface-active characteristics. It is commonly used in food, cosmetics, and pharmaceutical industries as a co-surfactant or emulsion^9,37^. Lecithin also functions as a biogenic mobilizing agent that is commonly used to wash sand contaminated with fuel oil^38^. Both natural and anthropogenic processes increase levels of lecithin. The excessive accumulation of lecithin in soil and water could stimulate the overgrowth of algae and increase the risk of eutrophication^4,39,40^. Lecithin is decomposed only under extreme physical/chemical conditions, e.g., high temperature, extreme pH, dehydration, freezing, chilling, and high mineral concentrations^41,42^. Therefore, it is necessary to develop eco-friendly strategies to control lecithin levels.

In this study, we compared the abilities of *Acinetobacter* (PSB) and *A. niger* (PSF) to break down lecithin. We also investigated the mineralization of the released P from lecithin. To identify the formation and composition of secondary minerals, we performed scanning electron microscopy (SEM) and energy dispersive spectroscopy (EDS) analyses.

## Results

### P Release from inorganic phosphates

*A. niger* increased the soluble P concentration in tricalcium phosphate (TCP)-amended PVK medium to 770.5 mg L^−1^ after incubation (Table 1). Accordingly, the pH of the medium in the presence of *A. niger* decreased to 3.5 (Table 1). As fluorapatite (FAp) (with lower solubility) was used as the P source, *A. niger* was still able to release soluble P. However, the maximum soluble P concentration in FAp medium was significantly lower (4.2 mg L^−1^) than when TCP was used as a P source. In addition, the pH of the medium decreased to 3.1 (Table 1).

**Table 1:** The highest levels of soluble P released by *A. niger* and *Acinetobacter* sp. detected after five-days incubation. TCP and FAp were added to the PVK medium as a P source.

Phosphorus sources	*A. niger*		*Acinetobacter* sp.	
	pH	Soluble P concentration (mg L^−1^)	pH	Soluble P concentration (mg L^−1^)
TCP	3.5 ± 0.1^a^ a^b^	770.5 ± 21.7 a	4.2 ± 0.0 b	402.3 ± 11.0 a
FAp	3.1 ± 0.3 a	4.2 ± 0.2 b	6.6 ± 0.0 a	0.9 ± 0.1 b

^a^Data were listed as mean ± S.E.

^b^Values followed by different letter (a–b) in the column are significantly different according to a Duncan’s Multiple Range Test at P < 0.05.

*Acinetobacter* sp. was less able to solubilize inorganic phosphates (from both TCP and FAp) than *A. niger*. In TCP medium, *Acinetobacter* sp. increased the soluble P concentration to 402.3 mg L^−1^ after five-days incubation. Accordingly, the pH of the medium pH decreased to 4.2, which was higher than that of medium containing *A. niger* (Table 1). The ability to release soluble P significantly decreased to 0.9 mg L^−1^ when FAp as P source, and the pH of the medium was close to neutral (Table 1).

### LMWOAs secreted by PSMs

**Figure 1:**
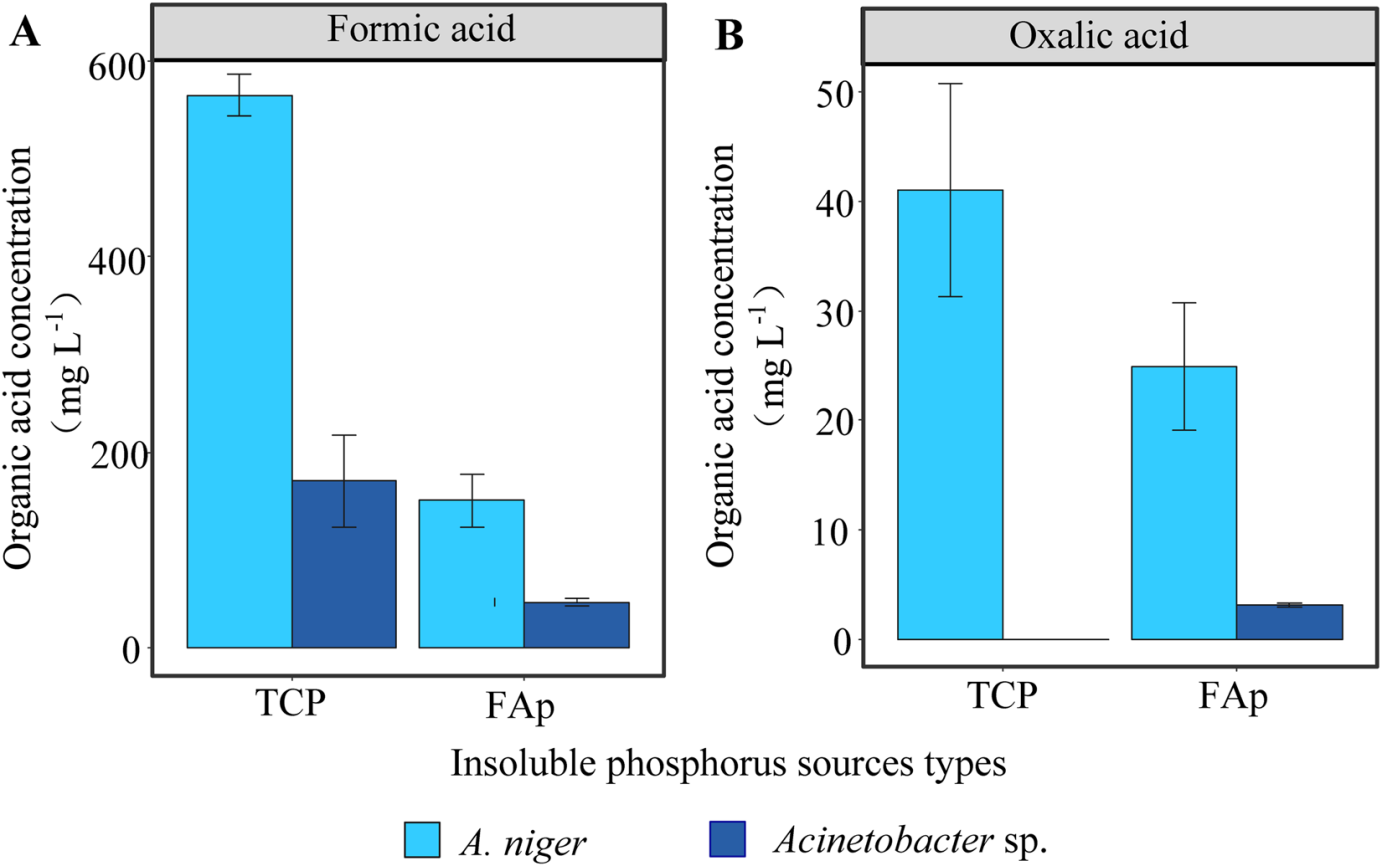
Concentrations of two major organic acids, formic acid (**A**) and oxalic acid (**B**), in medium containing *A. niger* and *Acinetobacter* sp. amended with TCP or FAp as a P source after five-days incubation. The columns show mean ± S.E.

*A. niger* secreted approximately 565.2 and 150.4 mg L^−1^ formic acid when TCP and FAp were used as P sources, respectively (Fig. 1A). *A. niger* also produced oxalic acid at levels of 41.0 mg L^−1^ in TCP medium and 24.9 mg L^−1^ in FAp medium (Fig. 1B). Oxalic acid is the primary compound secreted by *A. niger*. However, the oxalic acid concentration was extremely low in the presence of *A. niger* (Fig. 1B), probably due to the formation of calcium oxalate^34^.

Formic acid, a primary LMWOA, was present at relatively high concentrations containing *Acinetobacter* sp.; *Acinetobacter* sp. secreted 170.4 and 46.5 mg L^−1^ formic acid when TCP and FAp were used as P sources, respectively (Fig. 1A). However, the oxalic acid concentration in the medium was significantly lower in the presence of *Acinetobacter* sp. versus *A. niger* when either TCP or FAp was used as a P source (Fig. 1B). By contrast, the concentration of LMWOAs secreted by *Acinetobacter* sp. was always lower than that of *A. niger* (Fig. 1A,B).

### PSM - mediated lecithin breakdown

*A. niger* increased the soluble P concentration to 35.7 mg L^−1^ (17.9% of lecithin phosphorus) after five-days incubation (Table 2). We detected an obvious increase in soluble P concentrations beginning on the second day of incubation, which raised to 23.1 mg L^−1^. However, the rate of release of soluble P from *A. niger* was relatively low. The soluble P concentration was slightly higher than 30 mg L^−1^ until the fifth day of incubation (Table 2). The pH of the medium exhibited a downward trend throughout the incubation period. The lowest pH was 4.6 on the fifth day of *A. niger* incubation (Table 2).

**Table 2:** Soluble P concentrations and pH of medium containing *A. niger* and *Acinetobacter* sp. with lecithin as a P source during five-days incubation.

Days	pH		Soluble P concentration (mg L^−1^)	
	*A. niger*	*Acinetobacter* sp.	*A. niger*	*Acinetobacter* sp.
0	7.6 ± 0.1^a^ a^b^	7.6 ± 0.1 a	0.0 ± 0.0 c	0.0 ± 0.0 d
1	6.8 ± 0.0 b	6.8 ± 0.0 c	0.1 ± 0.1 c	3.2 ± 0.7 c
2	5.9 ± 0.0 c	6.9 ± 0.1 bc	23.1 ± 0.9 b	21.2 ± 0.2 b
3	6.0 ± 0.0 c	7.0 ± 0.0 bc	23.1 ± 3.3 b	30.6 ± 2.6 a
4	5.0 ± 0.1 d	7.0 ± 0.0 bc	28.0 ± 4.1 b	33.0 ± 3.3 a
5	4.6 ± 0.3 d	7.0 ± 0.0 b	35.7 ± 2.6 a	32.7 ± 2.5 a
S_1_^c^	135.5 ***^e^		0.8	
D_1_^d^	148.2***		161.5***	
S_1_ × D_1_	90.4***		0.0	

^a^Data were listed as mean ± S.E.

^b^Values followed by different letter (a–d) in the column are significantly different according to a Duncan’s Multiple Range Test at P < 0.05.

^c^S_1_, different inoculation treatments.

^d^D_1_, different incubation times (5 days).

^e^***, significantly different according to a two-way ANOVA at the 0.1% level with F value.

The soluble P concentration increased more rapidly in the presence of *Acinetobacter* sp. versus *A. niger* in the first three days of incubation, with the soluble P concentration in the medium reaching 30.6 mg L^−1^ (Table 2). In the presence of *Acinetobacter* sp., the soluble P concentration was elevated to 33.0 mg L^−1^ (16.5% of lecithin - P) after four-days incubation (Table 2). The pH of the medium decreased slightly on the first day of incubation, reaching a minimum of 6.8. However, in contrast to *A. niger*, the pH of the medium in the presence of *Acinetobacter* sp. increased slightly during the subsequent period, reaching 7.0 on the third day of incubation (Table 2).

### Changes in phosphatase activity in the PSMs

**Figure 2:**
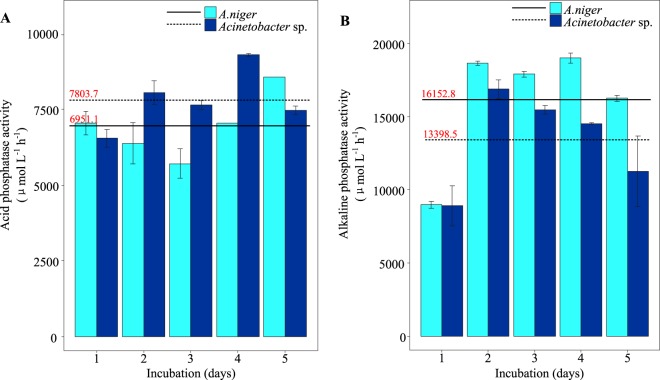
Dynamic changes in acid phosphatase (**A**) and alkaline phosphatase (**B**) activity in lecithin-amended PVK medium inoculated with *A. niger* and *Acinetobacter* sp. during a 5-day incubation period. The X-axis represents the days of incubation, and the Y-axis represents the enzyme activity level (phosphatase activity expressed in μmol L ^−1^ h^−1^). The solid lines represent the average enzyme activity of *A. niger*, and the dash lines represent the average enzyme activity of *Acinetobacter* sp.

Both *A. niger* and *Acinetobacter* exhibited acid and alkaline phosphatase activities (Fig. 2). Alkaline phosphatase activity was significantly higher than acid phosphatase activity in both *A. niger* and *Acinetobacter* sp.

The maximum acid phosphatase activity was slightly lower in *A. niger* than in *Acinetobacter*, reaching 8563.0 μmol L^−1^ h^−1^ after five-days incubation (Fig. 2A). The acid phosphatase activity of *A. niger* was significantly higher after the third day of incubation. The alkaline phosphatase activity for *A. niger* increased rapidly, with the highest activity (18,990.1 μmol L^−1^ h^−1^) detected on the fourth day of incubation (Fig. 2B). The average acid and alkaline phosphatase activities for *A. niger* were 6951.1 and 16,152.8 μmol L^−1^ h^−1^, respectively, during the incubation period.

In *Acinetobacter* sp., the highest acid phosphatase activity (i.e., 9300.0 μmol L^−1^ h^−1^) was detected on the fourth day of culture (Fig. 2A). By contrast, the alkaline phosphatase activity of *Acinetobacter* sp. rapidly peaked at 16,874.1 μmol L^−1^ h^−1^ on the second day of culture and gradually decreased thereafter (Fig. 2B). However, acid phosphatase activity was relatively steady. The average acid and alkaline phosphatase activities of *Acinetobacter* were 7803.7 and 13,398.5 μmol L^−1^ h^−1^, respectively.

Pearson’s correlation analysis revealed that the soluble P concentration was positively association with alkaline phosphatase activity for both *A. niger* (R^2^ = 0.3, P < 0.05) and *Acinetobacter* (R^2^ = 0.2, P < 0.05). There was also a significant positive correlation between acid phosphatase activity and soluble P concentration in *Acinetobacter* (R^2^ = 0.3, P < 0.05); however, this correlation was not significant in *A. niger*.

### P mineralisation by Ca cations

**Figure 3:**
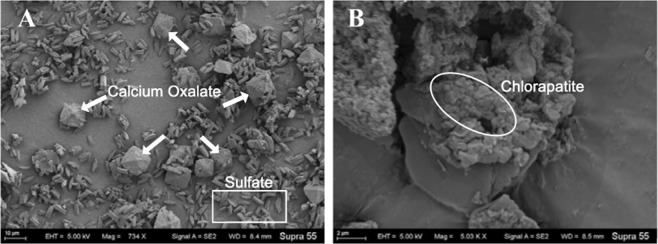
SEM images of precipitates formed after the addition of CaCl_2_ from supernatants of *A. niger* (**A**) and *Acinetobacter* sp. (**B**) cultures in lecithin medium after 5 days of incubation. The arrows and rectangle indicate the main forms of precipitates in *A. niger*, and the oval indicates the main form of precipitate in *Acinetobacter* sp.

Precipitates formed in the supernatants of *A. niger* and *Acinetobacter* cultures after incubation in lecithin, followed by the addition of CaCl_2_ (Fig. 3). In *A. niger*, the crystals showed a dipyramidal-prismatic shape, with sizes ranging from 2 to 10 μm (Fig. 3A). These crystals were likely composed of calcium oxalate based on their morphology, according to Li *et al*.^34^. EDS indicated that the major elements in the dipyramidal-prismatic crystals from *A. niger* were Ca, C, and O (Fig. S1A), confirming that they were composed of calcium oxalate^34,43^. We also observed many tandem rhomboid-like crystals in the precipitates. These crystals were likely sulphate minerals, as SO_4_^2−^ is present in PVK medium^44^.

The precipitates from *Acinetobacter* sp. included large aggregates (Fig. 3B), which were markedly different from the particles in *A. niger* shown in Fig. 3A. Higher-resolution imaging of the precipitates showed that these particles were primarily spherical and spheroidal. These shapes are identical to those of nano-chlorapatite^45^. The aggregated appearance of the particles could be attributed to aggregation during freeze-drying of the precipitates^45^. EDS further confirmed the abundance of Ca, Cl, and P on the surfaces of these particles, which are the major components of chlorapatite (Fig. S1B).

## Discussions

*A. niger* was able to secrete abundant LMWOAs to solubilize inorganic phosphates (Table 1 and Fig. 1). However, the ability of *Acinetobacter* sp. to break down OP (by phosphatase) was comparable to that of *A. niger* when using lecithin as a model OP compound (Table 2 and Fig. 2). Furthermore, to achieve P removal, the addition of CaCl_2_ to lecithin medium caused mineralization of the released P. However, the stable mineral chlorapatite only successfully formed in *Acinetobacter* sp. medium depleted of P (released from lecithin).

*A. niger* and *Acinetobacter* sp. secreted LMWOAs such as oxalic and formic acids, which solubilized inorganic phosphates at varying degrees (Fig. 1). These results are consistent with previous literature^34,46,47^. The greater ability of *A. niger* to solubilize phosphates could be attributed to the production of oxalic acid, as oxalic acid (pK_a_ = 1.4) is a strongly acidic LMWOA^34,48,49^. Additionally, oxalic acid can form secondary precipitates with the Ca^2+^ released from TCP and FAp, i.e., calcium oxalate. This results in lower concentrations of oxalic acid in *A. niger*. By contrast, the ability of *Acinetobacter* sp. to solubilize inorganic phosphate is primarily mediated by weak formic acid.

Phosphatase is an important extracellular enzyme in OP breakdown and plant P acquisition^2,6,7,50,51^. *Acinetobacter* exhibited higher acid phosphatase activity than *A. niger* during the 3 days in the middle of the incubation period (Fig. 2A). *Acinetobacter* sp. also showed considerable alkaline phosphatase activity, although it was lower than that of *A. niger* (Fig. 2B). Meanwhile, the soluble P concentration was higher in medium containing *Acinetobacter* versus *A. niger* beginning on the third day of incubation (Table 2). In addition, the pH levels of the culture medium of *A. niger* and *Acinetobacter* sp. were similar to the optimum pH (6.5) for acid phosphatase. Therefore, the contrast of the secretion of acid phosphatase might cause such difference of P release. Alkaline phosphatase is also highly efficient in OP decomposition, as reported in studies regarding sludge, but more active in alkaline environment^52^. Therefore, even the alkaline phosphatase could contribute to lecithin breakdown, but cannot exert its ideal function in this study.

Apatite is a stable P-containing mineral^53,54^. Here, chlorapatite, a variety of apatite, formed more readily in the supernatant from *Acinetobacter* sp. than from *A. niger* when cultured in the presence of CaCl_2_ (Fig. 3A). However, *A. niger* failed to help form chlorapatite, as this compound does not form readily in an acidic environment, according to Eq. (1)^45,55^. The pH of *A. niger* was significantly lower than that of *Acinetobacter* sp. after incubation (Table 1). Cl^−^ promotes pyromorphite formation at soluble P concentrations greater than approximately 10^−12^ mM, especially in a neutral environment^44^. Thus, stable chlorapatite could form in a Ca^2+^-PO_4_^3−^-Cl^−^ solution system in a neutral environment according to Eq. (2). Moreover, high levels of calcium oxalate were detected in *A. niger*, confirming that *A. niger* secretes oxalic acid, resulting in a drop in the pH of the medium (Fig. 3B). Therefore, *Acinetobacter* sp. are more suitable than *A. niger* in OP pollutant remediation under neutral conditions, as these conditions favour P precipitation^56^.

1 $${{\rm{Ca}}}_{{\rm{5}}}{({{\rm{PO}}}_{{\rm{4}}})}_{{\rm{3}}}{\rm{Cl}}+{{\rm{6H}}}^{+}\to {{\rm{5Ca}}}^{2+}+{{\rm{3H}}}_{{\rm{2}}}{{{\rm{PO}}}_{{\rm{4}}}}^{-}+{{\rm{Cl}}}^{-}$$

2 $${{\rm{5Ca}}}^{2+}+{{{\rm{3PO}}}_{{\rm{4}}}}^{{\rm{3}}-}+{{\rm{Cl}}}^{-}\to {{\rm{Ca}}}_{{\rm{5}}}{({{\rm{PO}}}_{{\rm{4}}})}_{{\rm{3}}}{\rm{Cl}}\,\downarrow $$

Many OP removal techniques, such as UV-LED/TiO_2_ photocatalytic (PC) and photoelectrocatalytic (PEC) technology, were usually high-cost^9^. In addition, the removal techniques of inorganic P, such as P adsorptions by bauxite residue and ferric chloride, could induce side effects to aquatic organisms^57,58^. The typical PSB, *Acinetobacter*, is a beneficial plant growth-promoting microorganism, and widely present in water bodies and soil^24,25,59–62^. Moreover, *Acinetobacter* sp. usually display a faster rate of reproduction than PSF^44^. Therefore, PSB could be a potential sustainable “biomaterial” in decomposing OP. Previous studies have proposed that there were several eco-friendly methods to remove the inorganic P by adding Mg^2+^ and Ca^2+^ to form struvite and hydroxylapatite^13,56^. A combination of PSB and CaCl_2_ successfully decreased the risk of eutrophication caused by OP in water bodies, and the OP degradation efficiency was about 16.5%, which is comparable to that of PC and PEC techniques^9^. Furthermore, Ca^2+^ and Cl^−^ are common elements in soil systems, which will not cause serious environmental pollution^15,63,64^.

## Conclusions

This work demonstrated that *Acinetobacter* (PSB) has a better potential for OP removal than *A. niger* (PSF). *A. niger* and *Acinetobacter* sp. were equally able to decompose OP (~30 mg L^−1^) after incubation. The ability of *A. niger* to solubilise TCP and RP was 1.9- and 4.6-fold greater than that of *Acinetobacter*, respectively, as it secretes a variety of LMWOAs. However, calcium oxalate was detected in the supernatant of medium inoculated with *A. niger* after the addition of CaCl_2_ because the environment was weakly acidic (final pH of 4.6). However, stable chlorapatite can be formed in the presence of Ca^2+^ and Cl^−^ in a neutral solution with addition of *Acinetobacter*. These findings deepen our understanding of OP decomposition using PSMs.

## Methods

### Strain selection and medium preparation

*Acinetobacter* sp. (CGMCC No. 13078) was isolated from cow manure provided by Qinbang Organic Fertilizer Company in Nanjing City, Jiangsu Province, China. *A. niger* (CGMCC No. 15994) was isolated from fluvo-aquic soil collected from fallow farmland in the upper 20 cm of soil in Nanjing, Jiangsu Province, China (118°66′E, 31°92′N) in April, 2017. Pikovskaya (PVK) medium (initial pH = 7.0) was used to determine the ability of *A. niger* and *Acinetobacter* sp. to solubilize inorganic phosphates^65,66^. The main components of PVK medium are 10.0 g/L glucose, 0.5 g/L (NH_4_)SO_4_, 0.03 g/L MnSO_4_·4H_2_O, 0.3 g/L KCl, 0.3 g/L MgSO_4_·7H_2_O, 0.03 g/L FeSO_4_·7H_2_O, 0.3 g/L NaCl, and 10 g/L P source. The levels of soluble P released by the two PSMs in PVK medium amended with 10 g tricalcium phosphate (TCP) (Nanjing Reagent Co. Ltd. of China, AR) or 10 g fluorapatite (FAp) (Taizhou Chemical Fertilizer Plant of China) as a P source after five-days incubation (Table 1). All experiments were performed with triplicate.

*Acinetobacter* was cultured in Luria-Bertani liquid medium, and *A. niger* was cultured in potato dextrose agar (PDA) medium. *Acinetobacter* sp. suspensions were collected by centrifugation at 16,099.2 g (4 °C) for 2 min. *A. niger* spore suspensions were collected by washing the spores on the surface of spore-forming PDA medium repeatedly with sterile water and filtering them through six types of gauze into an aseptic flask, according to the modified methods of Ren *et al*.^67^. Bacterial cells and fungal spores were adjusted to approximately 1 × 10^8^ CFU per mL using a haemocytometer.

### Detection of organic acid secreted by PSMs

To detect the LMWOAs secreted by PSMs, high-performance liquid chromatography (HPLC, Agilent 1260, USA) was used. For each sample, 2 mL of medium was collected from cultures in inorganic phosphate (for both TCP and FAp-amended PVK medium) after five-days of incubation. All samples were filtered through a 0.22-μm membrane prior to analysis. An RP-C_18_ chromatographic column (250 mm × 4.6 mm, 5 μm, Shimadzu, Japan) was used with a temperature of 30 °C. The mobile phase was 2.5% (NH_4_)H_2_PO_4_ (pH adjusted to 2.5 with phosphoric acid) with the chromatographic reagent methyl alcohol at the ratio of 99:1. The flow rate was controlled at 0.5 mL min^−1^, and the wavelength of detector was 210 nm.

### Quantifying the ability to breakdown lecithin

The ability of the PSMs to break down lecithin was quantified in the PVK medium amended with lecithin (0.2 g P L^−1^). A shaking culture experiment was then conducted using both PSMs. First, 1% suspensions (OD_600_ = 0.5) were inoculated into 50 mL lecithin-amended medium in triplicate. The cultures were shaken at 180 rpm at 30 °C for 5 days. During the incubation, 3-mL samples were collected daily under aseptic conditions and used to measure medium pH and soluble P concentration. The pH of the medium was then measured by a pH meter (FE 20, Mettler, German). The soluble P concentration was measured using the molybdate blue method and quantified spectrophotometrically at an absorbance wavelength of 880 nm.

To detect acid and alkaline phosphatase secreted by the PSMs during incubation, enzyme activity was analyzed using the disodium phenyl phosphate method (DPP)^68^. Briefly, 1 mL fermented lecithin medium was incubated with 4 mL universal buffer (pH = 6.5 for acid phosphatase and pH = 11 for alkaline phosphatase) and 1 mL 25 mM DPP for 1 h at 37 °C. Subsequently, 1 mL 0.5 M CaCl_2_ and 4 ml 0.5 M NaOH were added to terminate the reaction. The concentration of DPP was measured spectrophotometrically based on absorbance at 510 nm.

### Measurement of CaCl_2_ precipitation

Lecithin medium inoculated with the PSMs and incubated for 5 days was centrifuged at 16,099.2 g (4 °C) for 10 min to remove the microorganisms and insoluble residues. The supernatant was collected into a new flask. A rotary evaporator was used to concentrate the supernatant and increase the concentration of soluble P. CaCl_2_ (Nanjing Reagent Co. Ltd. of China, AR) was added to the supernatant at a Ca/P molar ratio of 100:1.

To achieve complete precipitation, the supernatant was shaken at 180 rpm at 30 °C for 1 h. The precipitates were collected by filtering through a 0.22-μm membrane and washed twice with sterile water to remove the liquid residue. To obtain dry powered samples, the precipitates were lyophilised at −40 °C for 24 h.

### Statistical analysis

Duncan’s multiple range test was used to analyse the differences between treatments. Two-way ANOVA and Fisher’s test were used to evaluate the pH and soluble P concentration of the medium after different periods of culture with different PSMs. All differences were considered to be significant at P < 0.05. All statistical analysis was conducted using R software version 3.3.2^69^.

## Supplementary information

Supplementary materials
