# Supplementary material for: Environmental fungi and bacteria facilitate lecithin decomposition and the transformation of phosphorus to apatite
Source: Sci Rep. 2019 Oct 25;9:15291. doi: 10.1038/s41598-019-51804-7 (PMC6814757; doi:10.1038/s41598-019-51804-7)
Supplement: Supplementary file 1 — Supplementary materials [file 41598_2019_51804_MOESM1_ESM.pdf]

Environmental fungi and bacteria facilitate lecithin decomposition and the transformation of phosphorus to apatite

Chunkai Li [a,1], Qisheng Li [a], Zhipeng Wang [a], Guanning Ji [a], He Zhao [a], Fei Gao [a], Mu Su [a], Jiaguo Jiao [a, b, *], Zhen Li [a, c, *], Huixin Li [a, b]

[a] College of Resources and Environmental Sciences, Nanjing Agricultural University, Nanjing, Jiangsu 210095, China

[b] Jiangsu Collaborative Innovation Center for Solid Organic Waste Resource Utilization, Nanjing, Jiangsu 210014, China

[c] Jiangsu Provincial Key Lab for Organic Solid Waste Utilization, Nanjing Agricultural University, Nanjing, 210095, China.

Corresponding to:

Dr. Jiaguo Jiao

College of Resources and Environmental Sciences,

Nanjing Agricultural University,

Nanjing, Jiangsu, 210095, China.

Tel. +86 13814049541; Fax: +86(25) 84399827

E-mail: jiaguojiao@njau.edu.cn

Dr. Zhen Li

Jiangsu Key Lab for Organic Solid Waste Utilization,

Nanjing Agricultural University,

Nanjing, Jiangsu, 210095, China.

Tel. +86(25) 84399827; Fax: +86(25) 84399827

E-mail: lizhen@njau.edu.cn

Precipitate characterization by scanning electron microscopy analysis

The precipitates that formed from supernatants prepared from *A. niger* and *Acinetobacter* sp. cultures containing $CaCl_2$ were subjected to crystalline morphological analysis by SEM. SEM was performed in a Carl Zeiss Supra 55 system with an acceleration voltage of 5 kV. The samples were sputter coated with gold for 5 min prior to SEM analysis. Semiquantitative analysis (collection time: 90 s) was performed using an Oxford Aztec X-Max 150 energy dispersive spectrometer.

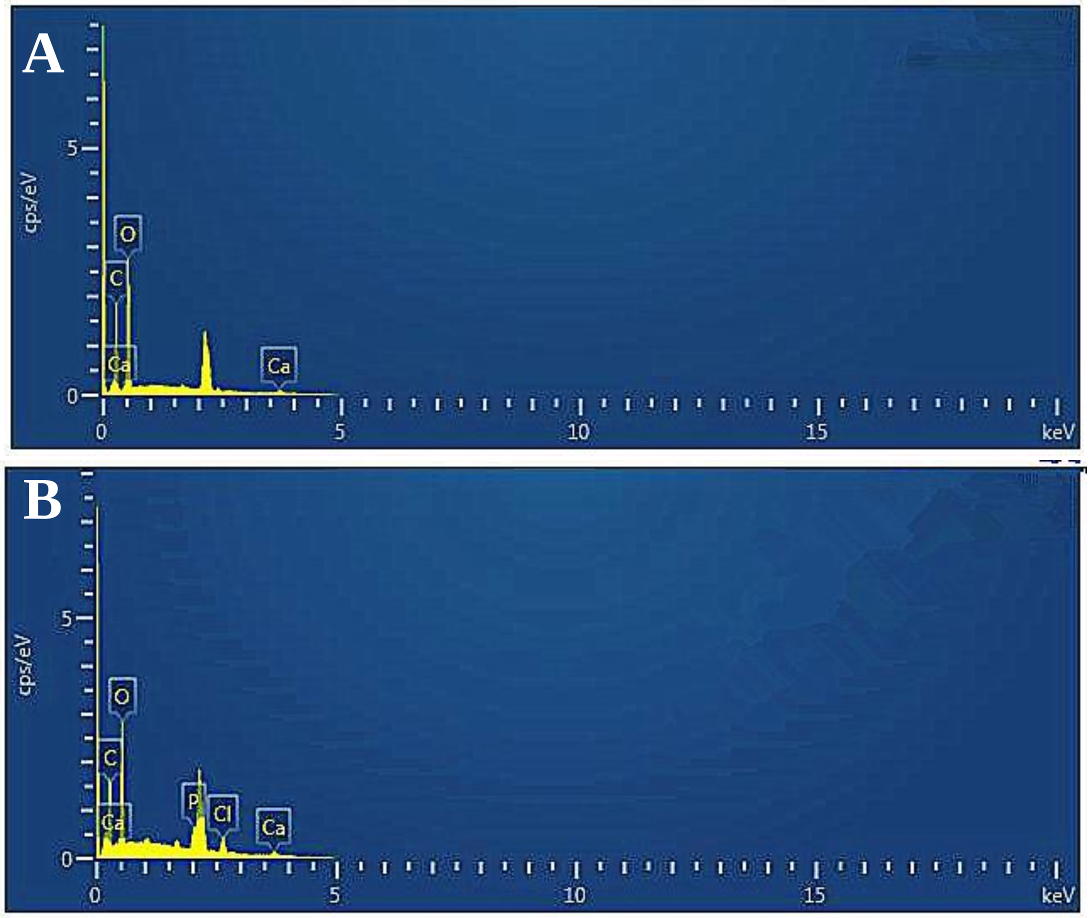

Supplementary Fig. 1. Elemental composition of precipitates formed in selected areas by adding $CaCl_2$ in *A. niger* (A) and *Acinetobacter* sp. (B), respectively.
